# p190RhoGAPs, the *ARHGAP35*- and *ARHGAP5*-Encoded Proteins, in Health and Disease

**DOI:** 10.3390/cells8040351

**Published:** 2019-04-12

**Authors:** Capucine Héraud, Mathilde Pinault, Valérie Lagrée, Violaine Moreau

**Affiliations:** 1 INSERM, UMR1053 Bordeaux Research In Translational Oncology, BaRITOn, F-33000 Bordeaux, France; capucine.heraud@inserm.fr (C.H.); mathilde.pinault1@gmail.com (M.P.); valerie.lagree-bringtown@u-bordeaux.fr (V.L.); 2 UMR1053 Bordeaux Research in Translational Oncology, University of Bordeaux, BaRITOn, F-33000 Bordeaux, France; 3 Equipe Labellisée Fondation pour la Recherche Médicale (FRM) 2018, F-33000 Bordeaux, France

**Keywords:** GTPases, GTPase-activating proteins, actin, RhoA, acto-myosin, cancers, neurological diseases

## Abstract

Small guanosine triphosphatases (GTPases) gathered in the Rat sarcoma (Ras) superfamily represent a large family of proteins involved in several key cellular mechanisms. Within the Ras superfamily, the Ras homolog (Rho) family is specialized in the regulation of actin cytoskeleton-based mechanisms. These proteins switch between an active and an inactive state, resulting in subsequent inhibiting or activating downstream signals, leading finally to regulation of actin-based processes. The On/Off status of Rho GTPases implicates two subsets of regulators: GEFs (guanine nucleotide exchange factors), which favor the active GTP (guanosine triphosphate) status of the GTPase and GAPs (GTPase activating proteins), which inhibit the GTPase by enhancing the GTP hydrolysis. In humans, the 20 identified Rho GTPases are regulated by over 70 GAP proteins suggesting a complex, but well-defined, spatio-temporal implication of these GAPs. Among the quite large number of RhoGAPs, we focus on p190RhoGAP, which is known as the main negative regulator of RhoA, but not exclusively. Two isoforms, p190A and p190B, are encoded by *ARHGAP35* and *ARHGAP5* genes, respectively. We describe here the function of each of these isoforms in physiological processes and sum up findings on their role in pathological conditions such as neurological disorders and cancers.

## 1. Introduction

Small guanosine triphosphatases (GTPases) of the Rat sarcoma (Ras) superfamily are molecular switchers of key signaling pathways. Within the Ras superfamily, members of the Ras homolog (Rho) family are crucial to regulate actin-based processes such as adhesion, migration, or cell division. The alteration of Rho pathways is implicated in various pathological conditions including cancer, cardiovascular diseases, or developmental disorders [1,2,3]. In physiological conditions, these key cellular processes need to be tightly controlled in space and time at the molecular level. Regulation of small GTPases occurs through nucleotide binding and hydrolysis allowing the well-known guanosine diphosphate (GDP)/guanosine triphosphate (GTP) cycle. Thus, the spatiotemporal regulation of this cycle is determinant for the accomplishment of signaling cascades and downstream cellular processes. The main regulators of the GDP/GTP cycle of Rho GTPases are the guanine nucleotide exchange factors (GEFs) and the GTPase-activating proteins (GAPs), allowing, respectively, GTPase activation or inhibition.

The p190RhoGAP paralogs, p190RhoGAP-A (p190A, glucocorticoid receptor DNA-binding factor 1 (GRF-1), or GRLF1, encoded by the *ARHGAP35* gene) and p190RhoGAP-B (p190B, encoded by the *ARHGAP5* gene), correspond to the two main GAPs regulating the Rho family of small GTPases. Both isoforms are large (≈1500 amino acid long) multidomain proteins with their catalytic RhoGAP domain at the C-terminus. In humans, p190A (Uniprot Q9NRY4) and p190B (Uniprot Q13017) share over 50% sequence identity, and especially 70% for their GAP domain, but only 45% for the middle domain, which is the most divergent domain between both isoforms. The *ARHGAP35* gene is conserved in several species like chimpanzee, Rhesus monkey, dog, cow, mouse, rat, chicken, zebrafish, fruit fly, mosquito, and frog, whereas the *ARHGAP5* gene is present in mouse, chicken, lizard, and zebrafish. *Drosophila* shows a single p190RhoGAP (Uniprot Q9VX32), which shares 54% identity with human p190A [4]. Amino acid residues of the human and mouse p190B proteins are approximately 97% identical [5]. Of note, in cellular studies, rat p190A (Uniprot P81128), which is over 98% identical to human p190A, is often used to address p190A function. 

Herein, we sum up their best-known cellular roles as regulators of Rho-dependent signaling processes such as cell adhesion, migration, invasion, and cytokinesis, even if Rho-independent implication of p190RhoGAPs emerges in the literature. We also address their important roles in development and in diseases.

## 2. p190RhoGAP Architecture

**Figure 1:**
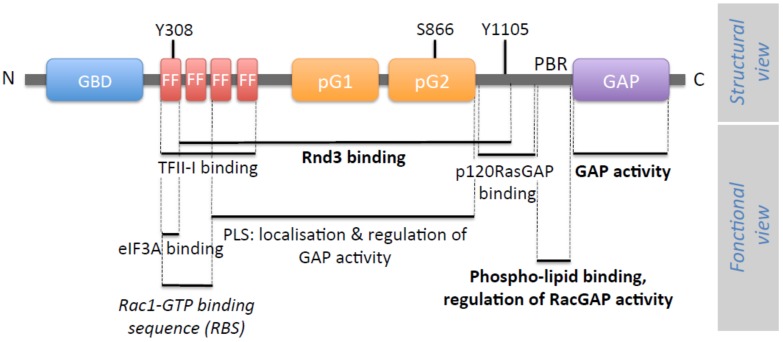
Structure of p190RhoGAP. The structural organization of p190RhoGAP is represented (structural view) and the corresponding function of each domain is indicated when determined (functional view). From its N-terminus, p190RhoGAP is composed of a guanosine triphosphate (GTP)-binding domain (GBD) (13–249) and four FF domains (FF) (251–533) that can bind TFII-I, eIF3A, or Rac1 proteins. In the middle domain, two pseudoGTPase domains, pG1 (592–767) and pG2 (766–958) have been identified. The protrusion localization sequence (PLS) (380–971) is implicated in the localization and the regulation of the function of p190A. A polybasic region (PBR) (1213–1236), mainly composed of basic amino acids, binds to acidic phospholipids. The GTPase activating proteins (GAP) domain (1259–1513) is located at the C-terminus end of the protein and is responsible for the GAP catalytic function of p190A and p190B. Identified residues involved in the function of p190RhoGAP are indicated on the figure (number of amino acids corresponds to the rat p190A protein sequence). For the functional view, bold indicates data obtained for both p190A and p190B. Italic indicates data obtained only for p190B, whereas basic font indicates data obtained for p190A.

**Figure 2:**
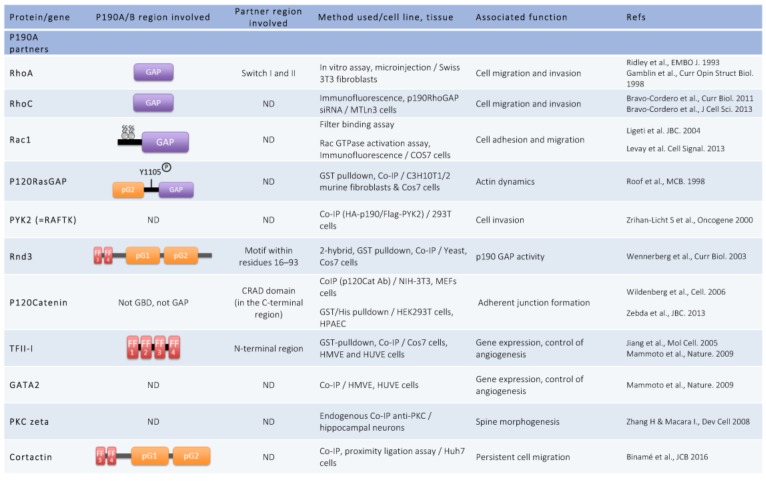

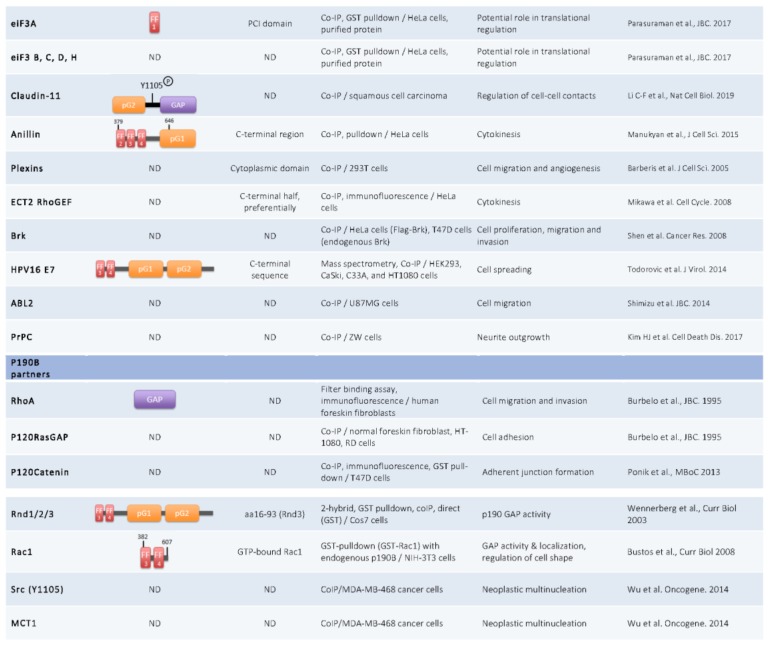
P190A and p190B partners. Co-IP: Co-immunoprecipitation; ND: Not Determined.

The two isoforms of p190RhoGAP share a common domain organization. Each domain has been studied either in a structural and/or functional approach (Figure 1). In addition to Figure 1 showing p190RhoGAP architecture, Figure 2 summarizes p190A and p190B partners.

The RhoGAP domain is located at the C-terminal end of p190RhoGAP proteins. Both p190A and B have mainly a catalytic activity towards RhoA [6,7] and p190A represents 60% of the RhoGAP activity in cultured fibroblasts [8]. Arg1283 was identified as the active site allowing efficient catalysis of RhoA-GTP [9]. By regulating RhoA activity, expression of p190RhoGAP in cells alters actin-based stress fibers formation, readout of RhoA function. Work from Dr. Ligeti’s laboratory demonstrates that both p190RhoGAP isoforms can modulate their substrate specificity by switching their GAP activity from RhoA to Rac1 upon interaction between a polybasic region (PBR) located near the GAP domain and phospholipids [10,11] (Figure 1). Like most of the RhoGAP proteins, besides their RhoGAP module, p190A and B contain several domains conferring plasma membrane-binding protein interactions, which modulate their GAP activity as well as provide them additional functions.

The N-terminal part of p190RhoGAPs displays a GTP-binding domain (GBD), which was recently reclassified as a class (ii) pseudo-GTPase domain, meaning a nucleotide-binding catalytically inactive GTPase [4]. Based on crystal structure and on biochemical data, Stiegler and Boggon showed that the N-terminal domain of p190A binds constitutively to GTP/Mg^2+^ but lacks intrinsic catalytic activity [4]. They further suggested that the role of GTP binding is not to act as a conformational regulator, but rather contributes to stabilizing the structure of the domain. This newly identified domain would so consist of a protein–protein interaction domain. In previous papers, GTP binding was reported to be regulated by a Src-mediated phosphorylation in this domain [12], but the impact of the lack of GTP binding on protein activity is not clear [13,14].

Closed to the GBD, four FF domains have been identified, each consisting of 50 amino acids motifs with two strictly conserved phenylalanines. These FF domains are usually found in nuclear RNA-regulating proteins, making p190RhoGAPs the only cytoplasmic proteins containing FF domains. This region has been implicated in interactions with proteins like the transcription factor TFII-I [15] or the translation preinitiation factor eiF3A [16] (Figure 2). These interactions have been reported to be regulated by phosphorylation. Phosphorylation of Tyr308 by PDGF Receptor within the first FF domain inhibits the interaction with TFII-I [15], but does not affect eiF3A interaction, which, itself, requires phosphorylation of Ser296 [16] (Figure 1 and Figure 2).

The former large “middle domain” defined by the region between the FF and GAP domains contains two newly identified cryptic GTPase-like domains which were termed pG1 and pG2 and classified as class (i) pseudo-GTPase domains described with no nucleotide-binding activity (and therefore no catalytic activity) [17]. pG1 and pG2, encompassing, respectively, residues 592–767 and 766–958 in p190A, constitute two evolutionarily conserved domains, indicating that they may contribute to necessary functions [18]. Intriguingly, based on molecular and cellular studies, we demonstrated that an overlapping region (residues 380–971), including two FF motifs, pG1 and pG2, is necessary to target p190A to actin-based protrusions such as lamellipodia but also to regulate the GAP activity. We further hypothesized that this region, named PLS for protrusion localization sequence, could be involved in an autoinhibitory interaction allowing a closed/inhibited form of the protein [19]. In light of these new structural results, one attractive hypothesis is that one of the pseudo-GTPase domains could bind in cis to the GAP domain to regulate its activity. However, this hypothesis remains to be tested. Within pG2, Ser866 appears as a key residue regulating p190A localization and function. In fact, mutation of this serine into phenylalanine results in a higher GAP activity that alters tumor cell migration [20].

Moreover, in this former middle domain, the regions encompassing residues 382–1007 in p190A/B and residues 382–607 in p190B were shown to interact, respectively, with the atypical Rho family member Rnd3 [21], and the active Rac1 [22], modifying p190RhoGAP activities. Thus, p190RhoGAP function appears tightly regulated by this large middle domain.

Finally, among the other residues important in the regulation and function of p190A, Tyr1105 was identified early as a Src phosphorylation site involved in the association of p190A with the Ras GAP protein, p120RasGAP and, consequently, in its recruitment to the plasma membrane [23,24,25,26] (Figure 2). Although phosphorylation of Y1105 is essential for complex formation, additional phosphorylation of Y1087 in p190A helps to stabilize the interaction [25]. Tyr1105 was also demonstrated to be substrate of other non-receptor tyrosine kinases such as Brk (breast tumor kinase) and ABL2 (v-Abl Abelson murine leukemia viral oncogene homolog 2)/Arg (Abelson-related gene) kinase resulting in the activation of p190RhoGAP [27,28].

## 3. p190RhoGAP Cellular Functions 

**Figure 3:**
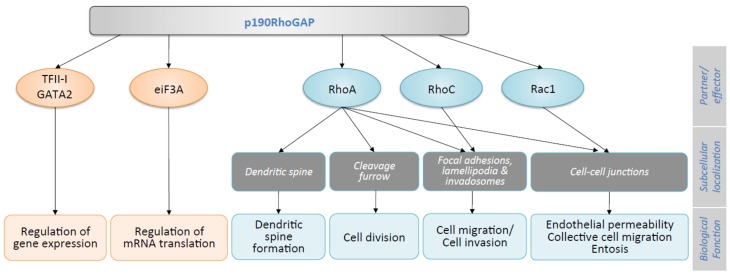
Cellular functions of p190A. P190A functions occurring independently of small GTPases are indicated in orange and those implicating the small GTPases are indicated in blue.

Due to their enzymatic activity consisting of an increased GTP hydrolytic rate and thereby an attenuation of the biological function of small GTPases, GAPs are often labeled as signal terminators. However, thanks to interaction with key partners, GAPs may also function as signal initiators and/or transmitters [29,30]. Function of both p190RhoGAP isoforms is intrinsically linked to RhoA regulation, even if more recently, few roles independently of RhoA were suggested (Figure 3). Of note, most of the data available, summarized in Figure 3, focused on p190A, while only few on p190B.

### 3.1. RhoA-Dependent Cell Mechanisms

P190RhoGAPs play their major role by inhibiting the GTPase RhoA and thus its downstream effectors, the Rho-associated coiled-coil-containing protein kinase (ROCK) and the formin mDia. Via ROCK, RhoA controls the phosphorylation of myosin light chain, thus cell contractility, and the cleavage of F-actin by inhibiting cofilin activity. Via mDia, RhoA promotes actin polymerization. Inhibition of RhoA by toxins or dominant negative inhibitors in vitro demonstrated essential functions of this GTPase in stress fiber formation, maintenance of cell–cell contacts, cytokinesis, and cell migration [2,31]. Consequently, p190RhoGAP activity leads to a major decrease in acto-myosin/contractility and thus impacts various RhoA dependent-cell mechanisms. This regulatory function depends on p190RhoGAP subcellular localizations and partner interactions (Figure 2 and Figure 3). Indeed, p190A accumulates in actin-rich structures where a regulation of RhoA is necessary. Localization of the protein in new adhesion sites [32], lamellipodia/ruffles [23], and podosomes/invadopodia [33,34] involved p190RhoGAP in cell migration and invasion. These well-described functions were reviewed elsewhere [19]. Other functions of p190A such as endothelial permeability, collective cell migration, entosis, cell division, and synaptic transmission are described herein. These roles are linked to p190A redistribution at either intercellular junctions [35], cleavage furrow [36], or dendritic spines [37].

#### 3.1.1. Cell–Cell Junctions and Endothelial Permeability 

Cadherin engagement to form adherens junctions was previously shown to inhibit RhoA activity via p190A [38]. This occurs through an interaction between p190A and p120-catenin at cell–cell junctions allowing p190A translocation to the membrane where it plays its role of GTPase regulator [35]. A domain in the C-terminus part of p120-catenin, named CRAD (catenin-RhoGAP-association domain) was recently identified as the minimal domain of interaction with p190A (Figure 2). In endothelial cells, depletion of the CRAD of p120-catenin affects p190A translocation to the membrane and is therefore responsible for an increase of RhoA and a decrease of Rac1 signaling, altering endothelial permeability [39].

#### 3.1.2. Cell–Cell Junctions and Collective Cell Migration

The sub-cellular localization of p190A at cell–cell junctions was further described to play a role in collective cell migration of squamous cell carcinoma [40]. Indeed, inhibiting RhoA activity is required to decrease actomyosin activity at cell–cell contacts and favor coordinated cell movement. More recently, this role was demonstrated to be important in the formation of circulating tumor cell clusters. An interaction between claudin-11 and p190A is required to downregulate RhoA at intercellular junctions in order to maintain stable cell–cell contacts during dissemination of these circulating tumor cells [41]. The RhoGAP activity at cell–cell junctions may also implicate p190B. Indeed, in breast cancer cells, RhoA activity at cell–cell contact is regulated by p190B through a complex with p120-catenin (Figure 2). Interestingly, this complex formation is modulated in response to matrix rigidity. This suggests that a cross-talk between cell–cell contact and cell-matrix adhesion is generated by the p190B/p120-catenin complex to spatially regulate RhoA activity in response to changes in tensional homeostasis [42].

#### 3.1.3. Cell–Cell Junctions and Entosis

Throughout its role on attenuation of cell contractility via RhoA down-regulation, p190A was recently implicated in the entotic mechanism. Entosis is described as a non-apoptotic cell death program for cancer cells, where one cell is able to ingest its living neighboring cell. This competition between tumor cells results from a difference in cell contractility between the invading (i.e., loser) and the engulfing (i.e., winner) cell [43]. On one hand, the winner cell exhibits high mechanical deformability, allowing changes in its shape and morphology. On the other hand, the invading cell accumulates actin and myosin at the cell cortex and the resulting mechanical tension drives the cell-in-cell invasion process. In this cellular mechanism, increased cell tension is regulated by Rho/ROCK following establishment of cell–cell adhesion [44,45]. Localization of p190A at cell–cell junctions in the invading cell allows the polarization of RhoA activity and the distribution of tension at the back, which promotes entosis. Accordingly, p190A is required for entosis, and downregulation of its expression reduced entosis [45]. 

#### 3.1.4. Cell Division

In eukaryotic cells, by its interaction with anillin, p190A localizes at the cleavage furrow of cells allowing a role of this protein during late mitosis [46,47]. Level of endogenous p190A is regulated during cell cycle leading to a decrease of the protein allowing cytokinesis completion. This transient decrease of p190A level during late mitosis is due to degradation of the protein via ubiquitination of the GBD domain [46]. P190A acts antagonistically with the RhoGEF ECT2 at the cleavage furrow where both proteins co-localize and probably interact [48]. A decrease in p190A level consequently regulates RhoA activity at the contractile ring, allowing completion of cytokinesis [36]. However, cells depleted of p190A failed to divide as well, as RhoA GTP induces increased contractility. Thus, p190A, by interacting with anillin, allows a fine regulation of the level of active RhoA necessary for cell division (Figure 2) [47]. However, p190A involvement in mitosis appeared more complex. In addition to its role in cytokinesis, p190A was recently implicated in regulating the mitotic spindle [49]. Interestingly, independently of its GAP activity, p190A is required for bipolar spindle formation in HeLa and RPE cells. This new role of p190A was demonstrated to occur through the regulation of the kinesin Eg5 and the kinase Aurora A activities [49].

#### 3.1.5. Dendritic Spines and Neuronal Morphogenesis

In addition to the roles described above, p190RhoGAP was implicated in the regulation of actin cytoskeletal rearrangements in cells from the central nervous system, with implications in brain functioning [50,51,52,53]. In *Drosophila*, p190RhoGAP was identified as essential for axon branch stability in body neurons, olfactory learning, and the memory center [53]. In fact, activation of RhoA or inactivation of p190A leads, in both cases, to axon branch retraction. The axon retraction pathway implicates Drok (*Drosophila* ROCK) and the myosin regulatory light chain [53]. Moreover, outgrowth of cellular extensions and formation of dendritic spines, which constitute actin-rich protrusions on dendrites essential for cognitive functions, are driven by actin cytoskeleton rearrangement and therefore under the control of RhoGTPases which are negatively regulated by p190A. In 2008, Zhang and Macara’s work on dendritic spines implicated the polarity protein PAR-6 and p190A in spine morphogenesis. They showed that, in hippocampal neurons, where p190A and RhoA are downstream effectors of the PKC/PAR6 complex, the catalytic GAP activity of p190A is necessary for spine formation, and depletion of p190A leads to a decrease of spine density [37]. p190A is also implicated in neuronal morphogenesis in the developing brain. Indeed, phosphorylation of p190A by Arg kinase leads to its interaction with p120RasGAP and regulates neuronal morphogenesis in mice postnatal brain. The p190/p120RasGAP complex was demonstrated to induce neuritogenesis in neuroblastoma cells [27]. Finally, in a recent study, p190A has been linked to the cellular prion protein (PrP^c^), a highly conserved glycosylphosphatidylinositol (GPI)-anchored membrane protein involved in neurite outgrowth (Figure 2). A p190A/PrP^c^ complex, which is impaired in the case of disease-associated mutants of PrP^c^, may be important to prevent prion-related neurodegeneration [54]. The impact of p190A in neuronal functions was further highlighted by p190A KO mice phenotypes (see paragraph 4) and by its involvement in neuronal diseases (Section 5.1).

### 3.2. RhoA-Independent Functions

Besides its RhoA-mediated functions, p190A also acts via other small GTPases such as RhoC and Rac1 or, as recently reported, via non-small GTPase pathways (Figure 3).

#### 3.2.1. RhoC/Rac1 Dependent Cell Migration and Invasion

In addition to RhoA, p190A demonstrated GAP activity towards RhoC and Rac1 (Figure 2). RhoC is a Rho family GTPase, which shares a high homology with RhoA [55] and promotes migration and invasion in many cancer cell types [56,57]. The activity of RhoC is regulated by both p190RhoGAP and p190RhoGEF at invadopodia [58] but also at protrusions at the leading edge of cells [59]. The spatial regulation of RhoC modulates protrusion formation by acting on actin dynamics and interfering with either of these proteins affect protrusion formation [60].

P190RhoGAPs display a GAP activity on Rac1 as well [11,61]. Based on in vitro experiments, the ability of p190RhoGAP to switch its GAP activity from RhoA to Rac1 was reported. In fact, due to the presence of some acidic phospholipids in the membrane, the activity of both p190A and p190B is modulated, leading to inhibition of RhoGAP activity and stimulation of RacGAP activity [11]. The switch between RhoGAP and RacGAP activity also depends on post-translational modification like prenylation of the GTPase [61]. The domain of interaction with acidic phospholipids was mapped and named PBR, consisting of a polybasic region composed of 24 amino acids close to the GAP domain in p190A (aa1213–1236) (Figure 1). Phosphorylation of Ser1121 and Thr1226 by PKC in PBR inhibits this interaction regulating thus the switch of GAP activity [62]. Similar to these in vitro experiments, under cellular conditions, p190RhoGAP exhibits RacGAP activity as well. Moreover, the PBR of p190A is necessary for the cellular RacGAP but not for the RhoGAP activity [10].

#### 3.2.2. Gene Expression and Protein Translation

In addition to its classical role of GAP protein acting as a GTPase inhibitor, p190A has been reported to interact with proteins involved in other cell mechanisms such as gene expression or mRNA translation, identifying p190A as a moonlighting protein.

P190A was implicated in gene expression regulation by its interaction with the multifunctional transcription factor TFII-I [63]. Intrigued by the FF domains present in p190A, the group of Pr. Settleman performed a pulldown approach to identify new partners of this domain. They thus identified the serum-responsive transcriptional regulator TFII-I as a specific interactor with the p190A FF domains (Figure 1 and Figure 2), and further demonstrated that these domains sequester TFII-I into the cytoplasm in a phosphorylation-dependent manner in fibroblasts [15]. P190A regulates the nuclear translocation and antagonizes the transcriptional activity of TFII-I. The cytoplasmic retention of TFII-I by p190A was further confirmed in human capillary cells where TFII-I governs vascular endothelial growth factor receptor 2 (*VEGFR2*) expression [64]. In these cells, p190A also inhibits the nuclear localization of the endothelial transcription factor GATA2. Through the regulation of both transcription factors, TFII-I and GATA2 that act antagonistically on *VEGFR2* expression, p190A was showed to control capillary network formation in vitro and retinal angiogenesis in mice [64]. 

In a similar pulldown approach, p190A was also reported to interact with eiF3A and other translation preinitiation factors (Figure 2). Indeed, five endogenous eIF3 subunits (eiF3A, B, C, D, and H) were identified to co-immunoprecipitate with endogenous p190A, but not p190B, in HeLa cells. This interaction leads to an incomplete and non-functional preinitiation complex. As eIF1A and other important translational preinitiation complex subunits were absent from the p190A complex, the authors hypothesize that p190A might affect the overall rate of mRNA translation by preventing the assembly of complete preinitiation complexes [16]. Given that no previous evidence had suggested a role for p190A GTPase substrates in the rate-limiting initiation step of mRNA translation, this new role of p190A may be independent of its GAP activity, even if this remains to be determined.

These two latter examples suggest that p190A may function as a sequestering molecule through interaction with various proteins in order to regulate key molecular processes such as gene expression and protein synthesis.

## 4. p190RhoGAP Function during Development

The respective roles of p190A and p190B isoforms in development were studied using *Arhgap35* and *Arhgap5* knockout in mice. Mice lacking a functional p190A protein die just after birth with several defects in neuronal development. The defects lead to aberrant neuronal morphogenesis and abnormalities in forebrain hemisphere fusion, ventricle shape, optic cup formation, neural tubes closure, and layering of the cerebral cortex [51]. Additional defects were identified in axon guidance and fasciculation [52]. Of note, these animals and mouse embryonic fibroblasts derived from them are not totally knocked out for p190A but instead display a non-functional form of the protein, lacking the GBD domain [51]. 

Mice lacking p190B also die after birth and exhibit alterations in cell and organism size [65]. As for p190A, p190B-lacking mice exhibit defects in nervous system development suggesting failure in axonogenesis, neuronal differentiation, and neurogenesis [66]. In p190B-deficient mice, a significant reduction in adipogenesis was observed [67] as well as an altered mammary ductal morphogenesis [68]. This latter defect due to p190B deficiency was extensively studied using an inducible system allowing the expression of p190B at different stages of mammary development. p190B overexpression during ductal morphogenesis or during pregnancy alters the terminal end buds (TEB) architecture, leading to disrupted ductal tree, and induces hyperplasic lesions, respectively [69]. As p190B, p190A was involved in mammary gland development but exhibits a distinct role in this process. P190A is implicated in cell adhesion within the TEB and its deletion alters the mammary epithelium differentiation [70].

Altogether, these findings obtained in murine models demonstrated that p190A and p190B play similar but still distinct roles in neuronal and mammary development.

## 5. p190RhoGAPs in Human Diseases

### 5.1. p190RhoGAPs in Neuronal Diseases

Due to the implication of p190A in neural development [51,66], few genome-wide and non-bias studies suggested a role of this protein in mental health disorders. 

Frontotemporal dementia (FTD) is one of the leading causes of dementia in young patients (<65 years old). Looking for genes potentially implicated in the development of FTD, Mishra and collaborators identified *ARHGAP35* as a candidate gene. Indeed, performing gene-based association studies on 3348 clinically identified FTD cases, they reported the novel genetic association of *ARHGAP35* with progressive non-fluent aphasia [71]. These data are further consolidated by the fact that *ARHGAP35* is strongly expressed in brain tissues, and more specifically, in the anterior cingulate cortex, which is one of the early affected regions in FTD patients [71].

Other studies involved p190A in stress-induced diseases. Stress exposure may lead to neuronal remodeling and structural plasticity. As p190A is localized at dendritic spines, the group of Koleske analyzed the role of p190A in the brain circuit using mice with reduced gene dosage of the protein [72,73]. They determined that p190A is involved in the behavior of mice and cellular response to the prolonged exposure to the major stress hormone, corticosterone [72]. They found that in neural systems, by inhibiting RhoA and subsequently, modulating cellular actomyosin contractility, p190A coordinates behaviorally adaptive outcomes such as mitigating vulnerability to stress hormone exposure, but also to drugs of abuse, as described hereafter. Indeed, underexpression of p190A was correlated with cocaine addiction in mice [73]. p190+/− mice in contrast to WT mice exaggerate locomotor activity and psychomotor sensitivity created by cocaine in link with a structural reorganization in neurons.

In humans, depression and, more precisely, suicide attempts are stress-induced diseases. With the aim to validate several genetic biomarkers associated with suicide attempts in patients with schizophrenia, Li and collaborators confirmed acid phosphatase 1 (ACP1) as a top predictor of suicide attempts [74]. This study also identified *ARHGAP35* as a gene whose expression is negatively correlated with APC1, that the authors linked with the transcription repressor of glucocorticoid receptor (NR3C1) to stress response. Altogether, these data suggest that p190A may be involved in phenomena of behavioral vulnerability and resilience throughout life in humans.

Alcohol consumption during pregnancy may lead to severe psychological and organic alterations known as fetal alcohol spectrum (FAS) disorders. Actin cytoskeleton and p190RhoGAP were identified as potential targets of ethanol in astrocytes [75]. Indeed, in in vitro models (HEK293T cells and primary cultures of newborn rat astrocytes), an increase of p190A and p190B activities was detected in response to ethanol chronic exposure. These data provide insights into the molecular mechanism responsible for the neurobehavioral deficiencies induced by alcohol.

In conclusion, p190RhoGAP may act as key factor in cellular response after chronic stress exposure induced by hormones or drugs. Through its role on cytoskeleton remodeling, p190RhoGAP may be linked to the pathogenesis of other stress-related and neurodegenerative diseases.

### 5.2. p190RhoGAPs in Cancer

Linked to the role of p190RhoGAP in cell cycle progression, cell migration and invasion, many papers described p190A and p190B implication in cancer progression. P190A was first described as a substrate of the oncoprotein c-Src, interacting with p120RasGAP, a key regulator of the Ras oncogene, and subsequently described to contribute to fibroblast transformation [76,77,78,79,80,81]. P190RhoGAP was reported to be involved in several cancer types based on data coming from either patient samples or cell lines (Table 1 and Table 2). Different types of alterations were reported concerning the mRNA or protein expression, the mutational or the phosphorylation status, and/or the GAP activity of p190A and p190B proteins. Most of the studies investigate p190A involvement, whereas only few focus on p190B. Hereinafter, we attempt to summarize data available on p190RhoGAP in the cancer field.

#### 5.2.1. ARHGAP35 as a Significant Mutated Gene in Cancer 

Several large-scale studies mapping somatic variants across thousands of tumors recently identified *ARHGAP35,* the gene-encoding p190A, as a new major cancer gene [82,83]. In 2013, *ARHGAP35* appears among 127 significantly mutated genes from the analysis of 3281 tumors across 12 cancer types, with the highest percentage in uterine corpus endometrial carcinoma [83]. These data were confirmed by Lawrence et al. in 2014. Exome sequencing analysis of almost 5000 samples from 21 tumor types identifies *ARHGAP35* as a candidate gene in cancer among 33 new genes. In this study, 2% of whole samples exhibit a mutation on *ARHGAP35* gene and this mutational status appears significant for five tumor types: Endometrial tumors (14%), lung squamous cell carcinoma (5%), lung adenocarcinoma (3%), head and neck cancer (3%), and kidney clear cell carcinoma (1%) [82]. The alteration of *ARHGAP35* gene in lung cancer was further confirmed by a more recent study dedicated to the identification of new driver genes in lung carcinogenesis. Indeed, *ARHGAP35* was highlighted as a new gene significantly altered in lung adenocarcinoma-lacking receptor tyrosine kinase–Ras–Raf pathway or other oncogene alterations [84]. Interestingly, in this study, *RASA1*-encoding p120RasGAP, a partner of p190A (Figure 2), was also found mutated in squamous cell carcinomas, suggesting that p190A/p120RasGAP pathway may be involved in lung carcinogenesis. Finally, searching for genetic drivers of Hürthle cell carcinoma of the thyroid, whole-exome sequencing also identified recurrent mutations in *ARHGAP35*, in addition to *NRAS*, *TP53*, *CDKN1A,* and *TERT* promoter [85]. Altogether, these data demonstrated that *ARHGAP35* is a new identified cancer gene. However, investigations should now be performed to correlate *ARHGAP35* mutations with clinicopathological features and prognosis of cancer patients.

To better understand p190A function during cancer development and progression, we may attempt to analyze mutation spectrum. Even if few hotspots may be distinguished (i.e., R997* in endometrial tumors [82] or E1273A in thyroid tumors or renal angiomyolipoma [85,86]), *ARHGAP35* mutations are observed all along the gene with nonsense mutations and frameshift deletions/insertions supporting a tumor-suppressor role for p190A (tumorportal website (http://www.tumorportal.org/)). At the protein level, as the GAP domain of the protein is localized at its C-terminus, these latter mutations are expected to behave as loss of function mutations, leading to a non-functional protein without GAP activity. This was confirmed by our study of the R997* nonsense mutation in vitro. Ectopically expressed p190A-R977* was unable to bind active RhoA, so to affect stress fiber formation [20]. Besides these mutations, many missense mutations were also reported all along *ARHGAP35* sequence. Even if some may have no impact on the protein activity as Y742H or R832Q found in endometrial tumors, we showed that some mutations localized in the pG2 domain (Figure 1) had a strong impact on the protein activity and on cell behavior [20]. Indeed, the point mutation S866F and the in-frame deletion del865-870 when ectopically expressed in tumor cells induce an increase of p190A activity and an alteration of directed tumor cell motility [20]. It is clear that the impact of such mutations remains to be explored in a closer way, but interestingly, these data allow us to hypothesize on a new mode of regulation of p190A, suggesting a potential auto-inhibitory folding of the protein [19]. Thus, even if, at first glance, cancer genome sequencing data support a tumor-suppressor function for p190A, more mechanistic studies about the impact of the cancer-associated mutations are required.

So far, no study has identified mutations in the p190B-encoding gene *ARHGAP5* in cancers. However, a genome-wide investigation into copy number variations of 221 colorectal adenomatous polyposis samples revealed recurrent alterations in three genes from the ARHGAP family, among which was *ARHGAP5*. A partial duplication and several somatic point mutations in this latter gene suggest a role for p190B in colorectal adenoma formation, and classify *ARHGAP5* as a candidate gene that may increase the risk of hereditary colorectal tumors [87]. This finding remains to be confirmed with other genetic studies.

#### 5.2.2. ARHGAP35 and ARHGAP5 as Tumor-Suppressor Genes 

Without analyzing *ARHGAP35* and *ARHGAP5* mutational status, several studies reported a decrease of expression of those genes in human tumors. 

Hepatitis viruses (HCV and HCB) are major risk factors for hepatocellular carcinoma (HCC), the main primary liver tumor. With the aim to focus on HCC lacking hepatitis virus background, Kurokawa and collaborators performed a PCR array gene profiling and observed a lower expression of p190A mRNA in HCC samples in comparison with non-tumor liver tissues [88]. Similarly, the expression of *ARHGAP5*, encoding p190B isoform, was found downregulated in invasive epithelial ovarian cancers [89]. 

Consistent with a tumor-suppressor function, *ARHGAP35* gene maps to the chromosomal region 19q13.3, which is also frequently deleted in gliomas, in pancreatic, ovarian, and thyroid tumors [90,91,92]. For gliomas, the tumor-suppressive role of p190A was studied in mice. In a model of oligodendrogliomas using PDGF (platelet-derived growth factor)-expressing retrovirus, co-expression of p190A GAP domain caused a decreased incidence of oligodendrogliomas compared with that observed with PDGF alone [92]. This finding is consistent with the fact that p190A is involved in the differentiation of glial cells [93]. 

In this context, the function of p190A often occurs through RhoA inactivation. Interestingly, RhoA inhibition by p190A or its C-terminus domain was suggested as a new approach in pancreatic cancer treatment. Indeed, overexpression of p190-RhoA chimera in human pancreatic cancer cells decreases the risk of developing liver metastasis in immune-incompetent mice [94]. As described above, by inactivating RhoA, p190RhoGAP activity leads to a major decrease in acto-myosin/contractility, inhibiting migration and/or invasion of cells. The activation of p190RhoGAP is mainly performed through phosphorylation by non-receptor tyrosine kinases such as Src, Brk, Blk (B lymphocyte kinase), ABL2/Arg kinase, and FAK (focal adhesion kinase), most of the time on Tyr1087 and Tyr1105 (Figure 1). These types of regulation of p190RhoGAP leading to alteration of tumor cell migration and invasion were demonstrated in various cancer cell types. In U87-MG glioma cells, the mediator of axon guidance semaphorin SEMA3F was shown to inhibit in vitro cell migration via the ABL2/Arg kinase-dependent activation of p190A and further inhibition of RhoA [95]. In prostate cancer cells, the microRNA miR-20a, which targets ABL2/Arg, was reported to be overexpressed [96]. In in vitro experiments, inhibition of miR-20a function led to an inhibition of migration and invasion of prostate cancer cells, via an activation of p190A [96]. In patients, this overexpression of miR-20a, which reduces p190A phosphorylation and its activation, correlates with a poor outcome. 

Several papers from Teixido’s laboratory focused on the involvement of p190RhoGAP in melanoma progression. They described p190A as a central molecule controlling melanoma cell invasion. Activation of p190A via phosphorylation by the tyrosine kinase Blk inhibits RhoA in melanoma cells [97]. Consequently, this signal decreased cell motility, invasion, and metastasis development in melanoma cellular and murine models [98,99]. They further demonstrated a key role of E-cadherin-based cell–cell junctions in this process via the interaction between p120-catenin and p190A [99]. 

Another interesting work from Parsons’ laboratory also suggests that p190A may function as a tumor suppressor by regulating apoptosis. Indeed, in breast cancer cell lines, ectopic expression of p190A induced cell death in a RhoA and caspase-dependent manner. Moreover, p190A is able to modulate the apoptotic response to docetaxel (used as chemotherapeutic drug) and increase the sensitivity of breast cancer cell to the drug [100]. More recently, work by Hansen’s laboratory suggests that p190A may alternatively play its tumor-suppressor function by promoting contact inhibition of cell proliferation through its cell–cell junction subcellular localization [101]. Indeed, they demonstrated that absence of p190A (but also p190B) is sufficient to alter contact inhibition of proliferation in epithelial cells cultured in matrigel. This function is dependent on the Hippo pathway, involving the translocation of YAP (yes-associated protein) to the nucleus and the downstream regulation of gene transcription [101].

Thus, altogether these data demonstrate that alterations of p190A arise in tumors from transformed cells of mainly epithelial origin. Thus, p190A through its numerous cellular functions is required to maintain a differentiated state of epithelial cells.

#### 5.2.3. ARHGAP35 and ARHGAP5 as Potential Oncogenes

In contrast with the data summarized above, overexpression and/or overactivity of p190RhoGAP proteins were also reported in a number of studies questioning the status of tumor suppressor of p190A and p190B. 

Overexpression of p190A was demonstrated in several cancer types at the protein or mRNA level and was proposed to influence the prognosis of patients. In osteosarcoma, high expression of p190A was correlated with poor outcome and associated with tumor dedifferentiation and increase of tumor size or metastasis risk [102]. Moreover, as a polymorphism in the 3′-UTR of *ARHGAP35* gene is associated with tumor size, tumor grade, tumor metastasis, and survival (overall and recurrence-free) of patients, p190A was proposed as an independent prognostic factor influencing survival of patients with osteosarcoma [103]. During colorectal cancer progression, from normal mucosa to liver metastasis through colorectal adenoma and primary carcinoma, p190A expression increased. Overexpression of p190A is thus correlated with poor prognosis in colorectal cancer [104]. In colorectal cancer cellular models, p190A activation together with p120RasGAP were shown to be important effectors of mutant *KRAS* [105]. In lung adenocarcinoma, p190A mRNA overexpression was associated with a hyperphosphorylation of the protein on Tyr1105 [106]. This hyperactivation of p190A leads to enhanced proliferation, migration, and invasion in EGFR-TKI cell line. This is in line with the poor disease-free survival of patients that harbor tumors with high levels of p190A mRNA [106]. At a glance, these data appear in clear contrast with the non-sense mutations recently found in lung adenocarcinoma [84], but p190A involvement may differ according to the tumor stage and/or mutational status. 

As described earlier, p190A was involved in the collective mode of cell migration of squamous cell carcinoma through its role in maintaining cell–cell junctions. P190A is required to decrease actomyosin activity at cell–cell contacts and favor coordinated cell movement [40]. More recently, this role was demonstrated to be important in the formation of circulating tumor cell clusters. Downregulation of RhoA by p190A at intercellular junctions is required to maintain stable cell–cell contact during dissemination of circulating tumor cells [41].

Moreover, in breast cancer, a depletion of p190A leads to a decrease of incidence of bone metastasis in mice model [107]. In vitro and in human breast cancer tissue samples, the non-receptor tyrosine kinase Brk is expressed, but not in benign lesions or in normal breast tissue, and is associated with breast carcinoma cell proliferation [108]. Interaction of Brk with p190A is required to increase Tyr1105 phosphorylation leading to RhoA inactivation and Ras activation in vitro. In breast cancer cells, this will result in an increase in cell migration and invasion but also an increase in tumor growth [28]. Thus, this study revealed that p190A acts downstream of Brk to promote breast tumor proliferation and migration. 

Similarly, p190B, which was reported essential for mammary gland development [68], was also suggested to play a role in mammary malignancy. Induction of p190B expression in mammary gland during pregnancy results in hyperplastic lesions in mice [69] and an increased level of p190B was detected in a subset of mutagen-induced mammary tumors [109]. More recently, overexpression of p190B was correlated with MCT1 (multiple copies in T-cell malignancy-1) expression in breast cancer. In vitro, MCT1-Src-p190B interaction gave rise to neoplastic multinucleation of breast cancer cells [110] that was suggested to favor tumorigenicity. This involvement of p190B was also studied in nasopharyngeal carcinoma and lung cancer where, respectively, miR-744 and miR-486-5 expressions correlated with p190B expression resulting in tumor progression and dissemination [111,112]. Several p190B loss-of-function studies in vitro and in vivo confirmed oncogenic role of p190B (Table 2). Depletion of p190B inhibits cell migration, cell invasion, tumor progression, and metastatic dissemination in different cell lines of lung cancer [112], breast cancer [110,113], hepatocellular carcinoma [114], and nasopharyngeal carcinoma [111].

Thus, in the oncogenic context described above, p190A and p190B may be predictive factors for the prognosis of different types of tumors.

### 5.3. p190A and Other Diseases

Few studies investigate p190A involvement in other diseases. Among them, low p190A activity was observed in idiopathic pulmonary fibrosis (IPF), which is a chronic lung disease characterized by a progressive and irreversible decline in lung function. RhoA activity is increased in IPF fibroblasts and associated with IPF phenotype such as expression of different markers like smooth muscle actin, collagen I, and fibronectin [115]. In glomerulocystic kidney defects, using a small-scale Nethyl-n-nitrosourea mutagenesis screen, Stewart et al. highlighted a point mutation L1396G in p190A leading to an alteration on cilia elongation and its involvement in renal developmental diseases [116].

## 6. Conclusions and Future Directions

Through Rho-dependent functions, p190RhoGAPs play a critical role in regulating actin cytoskeleton dynamics. Both isoforms p190A and p190B are ubiquitously expressed and co-exist in most cells. They share a common structural organization, often common partners, and similar subcellular localizations, but the interplay between both isoforms at the cellular level remains to be explored in detail. So far, data are in favor of distinct but overlapping functions that may or may not involve regulation of Rho family GTPases. Recent data highlighted new structural domains, i.e., pseudo-GTPases in p190RhoGAPs, and hypothesized a new mode of regulation, i.e., autoinhibitory folding for p190A, opening new paths to explore. Structural, combined with functional, studies would permit us to better understand the molecular mechanisms underlying the function of p190RhoGAPs. 

Their involvement in pathologic disorders is important to address to determine whether their signaling may be useful in a therapeutic approach. As described herein, p190RhoGAPs are suggested to play a role mainly in mental health disorders and in tumorigenesis. 

Aberrant regulation of Rho GTPase activity and corresponding effector pathways is described to be a hallmark of human tumors. However, the Rho GTPase signaling network is complex. For negative regulators as GAPs, the general trend is toward a reduced expression of GAPs [117]. Nevertheless, the emerging picture for p190RhoGAP is much more complex, probably dependent on the environment and on both tumor type and progression status. Study of cancer-associated mutations by using genome editing would contribute to getting clues about the role of p190RhoGAP in tumorigenesis. This may lead to unexpected functions and regulations of p190RhoGAP.

## Figures and Tables

**Table 1:** P190RhoGAP’s deregulation in cancers. This table lists data on p190A and 190B obtained from patients. Abbreviations: T, tumoral; NT, non tumoral; nd, not determined; IHC, immunohistochemistry; FISH, fluorescence in situ hybridization.

Cancer Type	Nb of Tumor/Control	Expression	Detection	Source of Deregulation	Output	References
p190A						
**Cancer**	3281 T (12 tumor types)	nd	Exome sequencing	Mutations	ARHGAP35 significantly mutated in cancer	Kandoth et al. Nature 2013
	4742 T (21 tumor types)	nd	Exome sequencing	Mutations: 83 missense, 38 nonsense, 16 frameshift and 2 splice site	ARHGAP35 significantly mutated in cancer	Lawrence et al. Nature. 2014
	191 gliomas + 115 gliomas + 37 ovarian cancers	nd	FISH assay	Locus rearranged/Chromosomal region frequently deleted in solid tumors	Alteration may be responsible of cancerogenesis	Tikoo et al. Gene. 2000
**Osteosarcoma**	247 T	Up	IHC	nd	Poor outcome (larger tumor size, tumor dedifferenciation, high metastatic risk, recurrence-free and overall survival)	Zhao et al. Tumor Biol. 2014
	247 T/428 NT	nd	TaqMan PCR	rs1052667 polymorphism (3′ UTR)	Association with osteosarcoma risk, grade and poor prognosis	Zhao et al. Bio Med Res Int. 2014
**Colorectal cancer**	114 T/NT	Up	qRT-PCR, IHC, WB	nd	Correlation between expression and grade Poor survival outcome	Li et al. Am Transl Res. 2016
	124 T	nd	PCR-SSCP	c.2834dupA > p.Asn946GlufsX11	Premature stop/Loss-of-function mutation	Ji Choi et al. Pathol. Oncol. Res. 2017
**Lung adenocarcinoma**	133 T/NT	Up	qRT-PCR, IHC	↗° phospho-p190A	Poor disease-free survival	Notsuda et al. Int J Oncol. 2013
	660 T/NT	nd	Exome sequencing, RNA seq	Missense & frameshift indel or nonsense mutations	ARHGAP35 alteration in oncogene-negative tumors	Campbell et al. Nature Genetics. 2016
**Hepatocellular carcinoma**	37 T/17 NT	Down	PCR array	nd	Potentially responsible of non-viral hepatocarcinogenesis	Kurokawa et al. J Hepatol. 2003
	44 T	nd	IHC	↗° Grp78 > FAK > phospho-p190A	Link between Grp78 and tumoral invasion	Su et al. BMC Cancer, 2010
**Hürtle Cell Carcinoma**	41 T/NT	nd	Exome sequencing	p.E1273A	Recurrently mutated in HCC (≈5% samples)	Gopal et al. Cancer cell. 2018
**Renal angiomyolipoma**	30 T	nd	Sanger sequencing	p.E1273A	Missense variant with uncertain functional effects	Giannikou et al. PLOS Genetics. 2016
**p190B**						
**Hepatocellular carcinoma**	47 T	Up	IHC	Positive correlation between the expression levels of CD147 and p190B	Role in HCC progression by regulation of cell movement through p190B and CD147	Chen et al. Cancer Cell Int. 2016
**Nasopharyngeal carcinoma**	52 T	Up	RT-qPCR, WB	↗°miR-744 > ↗°p190B	Tumor progression	Fang et al. Oncotarget. 2015
**Invasive epithelial ovarian cancer**	18,736 T (10,316 of serous histology) / 26,138 NT	Down	eQTL	nd	G allele of AKAP6 rs927062 correlated with reduced expression of ARHGAP5	Earp et al. PLoS ONE 2018
**Lung cancer**	54 T	Up	IHC, qRT-PCR	Inverse correlation between miR-486-5p and ARHGAP5 expression	Positive correlation of p190B expression and cancer stage and lymph node metastasis	Wang et al. Oncogene. 2014
**Colorectal adenomatous polyposis**	221 T / 531 NT		Genome-wide analysis of germline CNV, NGS	Somatic point mutations/Partial duplication	One of 11 promising causative candidate genes	Horpaopan et al. Int. J. Cancer. 2015
**Breast cancer**	120 T / 7 NT	Up	qPCR array	Positive correlation between MCT-1 expression and ARHGAP5 expression in tumors	MCT-1 controls cancer cell dissemination and progression through Src/p190B signaling pathway	Wu et al. Oncogene. 2014

**Table 2:** Cellular or molecular effects of p190RhoGAP deregulation in cancers. This table lists functional deregulations of p190A or p190B in cancer studied in in vitro and in vivo experiments. Abbreviations: o/e, overexpression; nd, not determined.

Cancer Type	Cell Lines	Loss or Gain of Function Experiment	Output on p190A Activity	Output on Cell Proliferation	Ouput on Cell Migration/Invasion	Other Output	References
p190A							
**Osteosarcoma**	MG63	Loss (si p190A)	nd	Decrease	nd	Increase of apoptosis	Zhao et al. Tumor Biol. 2014
**Breast cancer**	MDA-MB-231	Gain (plasmids p190A)	Increase Brk/p190A interaction, phosphorylation of p190A and p190/p120 complex formation	Increase	Increase	Increase tumorigenicity (mice model)	Shen et al. Cancer Res. 2008
	MDA-MB-468, MCF10A	Gain (plasmids)	nd	nd	nd	Induction of caspase- and Rho-dependent apoptosis	Ludwig and Parsons. Genes & Cancer. 2011
**Lung adenocarcinoma**	A549, LK87, PC-14, H1975	Loss (si p190A)	Decrease p190/p120 complex formation, Ras inactivation, Rho activation	Cell cycle arrest	Decrease	Inhibition of Ras pathway	Notsuda et al. Int J Oncol. 2013
**Glioma**	PDGF/Gtv-a cells	Gain (WT GAP domain)	nd	Inhibition of PDGF-induced proliferation	nd	nd	Wolf et al. Genes Dev. 2003
**Renal cancer**	RCC, 786-O	Loss (si p190A)	nd	nd	nd	Restore fibronectin matrix assembly	Feijoo-Cuaresma. J Biol Chem. 2008
**Melanoma**	BLM human melanoma cells	Loss (si p190A)	p120ctn allows p190-RhoA interaction and RhoA activation	nd	E-cadherin melanoma transfectant invasion	nd	Molina-Ortiz et al. J Biol Chem. 2009
	LOX melanoma cells	Loss (injection of p190 antibody)	nd	nd	Decrease of matrix degradation	No alteration of cell adhesion	Nakahara et al. J Biol Chem. 1998
**Squamous cell carcinoma**	10 SCC cell lines and a primary HNSCC	Loss (sh p190A)	nd	nd	Dissociation of cancer cells	nd	Li et al. Nat Cell Biol. 2019
**Pancreatic cancer**	AsPC-1, PANC-1	Gain (p190-RhoA chimera)	Inhibition of RhoA activity (RhoB and RhoC)	No significant effect	Decrease of cell invasion, metastatic formation	nd	Kusama et al. Cancer Sci. 2006
**Human Papillomavirus**	HaCat, CaSki, C33A, U2OS, HT1080	Gain (plasmids p190A-HA)	Interaction MD p190A & CR3 of HPV E7	nd	nd	Decrease actin stress fiber formation, cell spreading	Todorovic et al. J Virology. 2014
**Cancer**	Huh7, MDA-MB-231, MEF	Gain (plasmids p190A-HA)	ΔPLS, S886F and Δ865-870 increase RhoGAP activity	nd	Alteration of cell migration directionnality	Defect in cell protrusion localisation and lamellipodia persistence	Binamé et al. J Cell Biol. 2016
	MDCK cell lines	Loss (sh p190A)	nd	Loss of CIP	nd	Nuclear translocation of YAP, rescue repression of gene transcription of Hippo pathway	Frank et al. J Cell Biol. 2018
**p190B**							
**Breast cancer**	MDA-MB-468	Loss (si p190B)	Interaction between p190B and MCT1 and also Src	Decrease acytokinetic division and neoplastic multinucleation induced by MCT1	nd	Inhibition of tumor growth	Wu et al. Oncogene. 2014
	MCF7-10A, rats	Gain (o/e p190B)	Overexpression of p190B in some murine mammary tumors	nd	nd	Actin cytoskeleton reorganization, loss of adhesion	Chakravarty et al. Cell Growth Differ. 2000
	Neu mice	p190B haploinsufficiency	Affect Rho signaling pathways	nd	nd	Inhibition of tumor initiation and progression, increase tumor free survival	Heckman-Stoddard et al. Breast Cancer Res. 2009
**Hepatocellular carcinoma**	Huh7	Loss (si p190B)	Increase RhoA activity	nd	Increase cell spreading and cell migration	nd	Gen et al. Cancer Lett. 2009
	SMMC-7721, Huh7, HepG2	Loss (si p190B)	Increase of RhoA activity	nd	Decrease migration induced by CD147	nd	Chen et al. Cancer Cell Int. 2016
**Nasopharyngeal carcinoma**	NPC cell lines	Loss (si p190B)	nd	nd	Abrogates migration and invasion induced by miR-744	nd	Fang et al. Oncotarget. 2015
**Cancer**	MDCK cell lines	Loss (sh p190B)	nd	Loss of CIP	nd	Nuclear translocation of YAP, rescue repression of gene transcription of Hippo pathway	Frank et al. J Cell Biol. 2018

## Abbreviations

Abl2	v-Abl Abelson murine leukemia viral oncogene homolog 2
ACP1	acid phosphatase 1
Arg	Abelson related gene
Blk	B lymphocyte kinase
Brk	breast tumor kinase
CRAD	Catenin-RhoGAP-Association Domain
EGFR-TKI	epidermal growth factor receptor tyrosine kinase inhibitor
FAK	focal adhesion kinase
FAS	fetal alcohol spectrum
FTD	Frontotemporal dementia
GAP	GTPase activating protein
GBD	GTP-binding domain
GDP	guanosine diphosphate
GEF	guanine nucleotide exchange factor
GPI	glycosylphosphatidylinositol
GRF-1	glucocorticoid receptor DNA-binding factor 1
GTP	guanosine triphosphate
GTPase	guanosine triphosphate hydrolase
HCC	hepatocellular carcinoma
IPF	idiopathic pulmonary fibrosis
MCT1	multiple copies in T-cell malignancy-1
PBR	polybasic region
PDGF	platelet derived growth factor
PLS	protrusion localization sequence
PrPc	cellular prion protein
Ras	Rat sarcoma
ROCK	Rho associated coiled-coil-containing protein kinase
Rho	Ras homolog
TEB	terminal end buds
YAP	yes-associated protein
